# Building Health Promotion into the Job of Home Care Aides: Transformation of the Workplace Health Environment

**DOI:** 10.3390/ijerph14040384

**Published:** 2017-04-05

**Authors:** Naoko Muramatsu, Lijuan Yin, Ting-Ti Lin

**Affiliations:** 1 School of Public Health and Institute for Health Research and Policy, University of Illinois at Chicago, Chicago, IL 60612, USA; lyin4@uic.edu; 2 College of Nursing and Institute for Health Research and Policy, University of Illinois at Chicago, Chicago, IL 60612, USA; tlin41@uic.edu

**Keywords:** workplace health, physical activity, health promotion, direct service workers, long-term services and supports, caregivers

## Abstract

Home care aides (HCAs), predominantly women, constitute one of the fastest growing occupations in the United States. HCAs work in clients’ homes that lack typical workplace resources and benefits. This mixed-methods study examined how HCAs’ work environment was transformed by a pilot workplace health promotion program that targeted clients as well as workers. The intervention started with training HCAs to deliver a gentle physical activity program to their older clients in a Medicaid-funded home care program. Older HCAs aged 50+ reported increased time doing the types of physical activity that they delivered to their clients (stretching or strengthening exercise) (*p* = 0.027). Almost all (98%) HCAs were satisfied with the program. These quantitative results were corroborated by qualitative data from open-ended survey questions and focus groups. HCAs described how they exercised with clients and how the psychosocial work environment changed with the program. Building physical activity into HCAs’ job is feasible and can effectively promote HCAs’ health, especially among older HCAs.

## 1. Introduction

Home care aides (HCAs) are non-medical paid caregivers who provide long-term services and support, such as routine housekeeping (e.g., laundry, grocery shopping, preparing meals) and personal care services (e.g., bathing, dressing). Typically middle-aged or older, low-income, ethnic minority women, HCAs constitute one of the fastest growing occupational groups in the United States, projected to add 458,100 jobs between 2014 and 2024 [1].

HCAs are similar to many other front-line service sector workers: HCAs receive limited workplace benefits such as work-site wellness programs and lack their own resources (e.g., time, money, opportunities) to make the best use of evidence-based health care and prevention strategies [2,3]. Like many other care workers who directly interact with their clients, HCAs face high levels of stress and occupational health risks (musculoskeletal injuries, exposures to needles and chemicals such as cleaning supplies, verbal and physical abuse) in their work environment [4,5] intertwined with stress experienced in their personal lives with challenging socioeconomic and community contexts [6,7,8].

Yet, HCAs’ work environment is unique. HCAs’ workplace is their clients’ private homes and thus it is difficult for managers or health promotion program staff to reach and intervene there. HCAs often work in isolation without direct supervision or peers in their workplace. Despite their regular contact with clients and intimate knowledge of their life styles and health conditions, HCAs lack opportunities to be part of their clients’ care team in current health care systems [2]. HCAs are hired to meet clients' needs for housekeeping and daily activity assistance, but not to play an active role to engage clients in practices that promote health and safety. This is a missed opportunity, not only to improve the quality of care for clients but also to empower HCAs with enhanced health literacy, and lifetime skills and competency to promote health for themselves, their families and communities.

Workplaces are increasingly recognized as important venues for promoting population health [9]. However, workplace health promotion programs are not equitably distributed [10,11]. HCAs’ employers, which range from individual clients (e.g., low-income seniors who receive Medicaid funds to hire their own family or relatives as HCAs) and small or mid-sized agencies to large multistate corporations, tend to focus on clients’ health and well-being. Employers generally lack awareness, resources, or incentives to implement health promotion or wellness programs for HCAs [12]. Little is known about how to make workplace health promotion programs appropriate and feasible for HCAs and other direct service workers.

To address this gap, we pilot-tested a health promotion intervention program that trained HCAs to deliver a safe, gentle physical activity program to their older clients as part of HCAs’ work in a Medicaid-funded home care program. The program aimed not only to enhance the function of home care clients, but also to empower HCAs with health literacy and concrete tools to promote health behavior changes in their clients and others. The program’s implementation process has been described elsewhere [13]. This current paper examined the transformation of HCAs’ work and health environment, focusing on the impact of the program on HCAs themselves. Two research questions were addressed: (1) Did HCAs’ own physical activity increase after the intervention? (2) How was the program received by HCAs?

## 2. Materials and Methods

### 2.1. Overview

**Figure A1:**
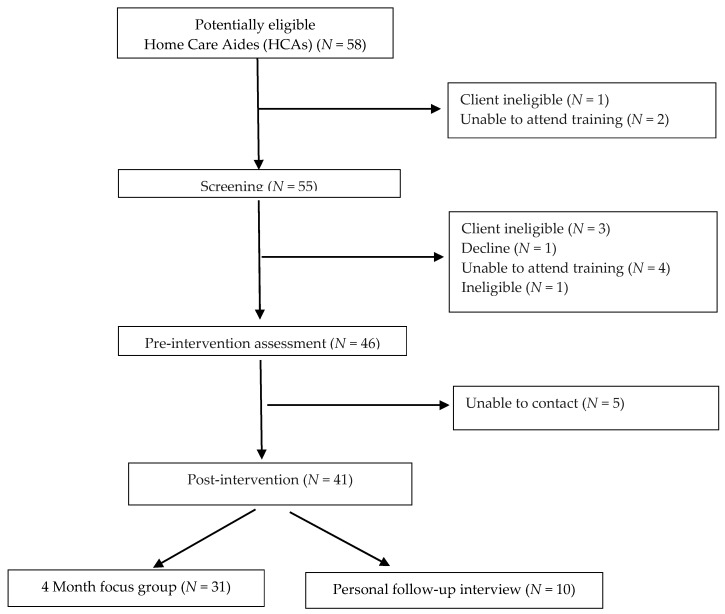
Flow diagram of home care aides recruitment and retention in Promoting Seniors’ Health with Home Care Aides: A Pilot.

A mixed methods approach was employed to evaluate the pilot program using a single group pre- and post-test design. The study participants consisted of 46 HCAs and their clients (54 pairs, with 4 HCAs caring for two enrolled clients). All 46 HCAs attended the training and completed self-administered questionnaires in group settings before the intervention. Thirty-one attended a follow-up training after 4 months and participated in a post-intervention survey and a focus group discussion, while 10 HCAs who could not participate in the follow-up training session were interviewed individually by the trained research interviewers. Five HCAs did not participate in the post-intervention survey, because the research staff could not reach them. HCAs who dropped out were older and had fewer years of home care experience than those who stayed in the program (see Figure A1 in Appendix A for a flow chart of HCA study participant recruitment and retention).

The quantitative data from the surveys were complemented by the qualitative data from: (1) HCAs’ responses to open-ended questions in the surveys and (2) focus groups. The study was approved by the Institutional Review Board at the University of Illinois at Chicago (Protocol #: 2012-1115). All participants signed written informed consent forms.

### 2.2. Research Context

Study participants were recruited from a large home care agency that provides in-home services to older adults eligible for the Illinois Department on Aging Community Care Program. This program provides home and community-based services to residents of Illinois aged 60+ with assets ≤$17,500 (excluding home, car, or personal furnishings) who have an assessed need for long term care. This state program is partly financed by the Medicaid waiver program, a rapidly growing program that is the main mechanism through which states finance home and community-based services for older adults with limited resources. Qualifications for HCAs included a high school diploma or general education diploma, one year of HCA work experience, or demonstration of continued progress toward a general education diploma. The state requires HCAs to attend 24 h of initial pre-service training before employment, and thereafter, a minimum of 12 h per calendar year of interactive in-service training approved by the provider agency. The participating home care agency is unionized, and thus their HCAs were represented by the labor union, SEIU Healthcare Illinois & Indiana.

### 2.3. Intervention

Healthy Moves for Aging Well is a low-cost physical activity program, developed by Partners in Care Foundation to safely enhance the activity level of home-bound older persons who are nursing home-eligible in a Medicaid program [14]. Based on evidence-based physical activity [15,16] and behavioral change theories [17], Healthy Moves has been endorsed by the Administration on Aging and National Council on Aging Evidence-based Prevention Initiative [18]. Healthy Moves involves a brief motivational enhancement and physical activity. Three gentle moves in a sitting position were included in the intervention: seated step-in-place (for lower extremity strength and aerobics), arm curls (for upper body endurance and strength), and ankle point & flex (for increased ankle flexibility and increased blood circulation to manage and prevent ankle swelling). Our study translated the original case manager-led Healthy Moves into a HCA-led program.

HCAs attended a 4-h training session led by the research staff. HCAs learned how to deliver the brief motivational enhancement and 3 chair-bound movements to the clients, and how to remind their clients to do Healthy Moves every day as part of regular home care visits. The training hours were counted towards state-mandated in-service training hours to facilitate HCA participation. HCAs attended one of the eight training sessions that were offered between August 2014 and January 2015 to accommodate waves of participant recruitment.

The intervention started immediately after the training. On the first home visit, HCAs assessed clients’ readiness for physical activity, had their clients set personally meaningful goals, and taught the 3 moves. On average the process took 27 min, ranging from 10 to 45 min. For the next 4 months, HCAs were asked to remind their clients of their personal goal and daily exercise routines as part of their regular home care, and to fill out a simple activity log. HCAs were asked to encourage clients to do the following moves every day: 15 arm curls two times (holding a 1 pound weight); ankle point & flex up to 30 s on each foot three times; and seated step-in-place up to 1 min.

### 2.4. Quantitative Data

#### 2.4.1. Measures

HCAs’ physical activity levels and related concepts were assessed by baseline and post-intervention surveys [19]. Participants were asked how much total time participants spent on each of the six different exercise categories (stretching or strengthening, walking, swimming, bicycling, using aerobics equipment, and other) during the past week (none, less than 30 min, 30–60 min, 1–3 h, and more than 3 h) [19]. Two additional physical activity-related concepts were measured at baseline and after the intervention. Readiness to act on regular exercise was measured by the Stages of Change short form (a single item) [20,21]. Exercise self-efficacy was measured by the abbreviated version of Self-Efficacy and Exercise Habits Survey (12 items, 5-point scale; Cronbach’s alpha = 0.86) [22].

In the post-intervention survey, HCAs were asked to rate the extent to which the program motivated them to be physically active and made them more physically active. HCAs evaluated two additional aspects of Healthy Moves: the training (four items; e.g., “increased my ability to motivate my client to be physically active”), and the extent to which HCAs used what they learned (2 items; one for their clients, the other for their family or friends). Five-point Likert scales were used (from “strongly agree” to “strongly disagree”). Finally, HCAs’ overall satisfaction with the program was assessed by a single question with four response categories (very satisfied to very dissatisfied).

#### 2.4.2. Analytic Strategies

Changes in HCAs’ physical activity and related items between pre- and post-intervention assessments were examined by Wilcoxon signed-rank tests for ordinal variables and two-tailed paired *t*-tests for continuous variables. HCAs’ evaluation of the program in the post-intervention survey was examined using descriptive statistics. Analyses were conducted by using Stata SE 13.1 (StataCorp LP, College Station, TX, USA).

### 2.5. Qualitative Data

#### 2.5.1. Open-Ended Survey Question

The post-intervention survey included a question on how satisfied HCAs were with the Healthy Moves program, followed by an open-ended question probing reasons behind HCAs’ level of satisfaction. HCAs wrote one to two sentence responses in the relevant space in the self-administered survey, which were subsequently transferred into an electronic file using REDCap. Almost all 31 HCAs were very satisfied (80.6%) or somewhat satisfied (16.1%) with the program. The one HCA who was not satisfied did not provide any qualitative information.

#### 2.5.2. Post-Intervention Focus Groups with HCAs

HCAs provided an in-depth description of their experiences with Healthy Moves in four focus group sessions attended by a total of 31 HCAs (*N* = 9, *N* = 5, *N* = 5, *N* = 12). Each one-hour focus group session started with an ice breaker (a brief introduction about HCAs, including the number of clients for whom they implemented the program, client’s age and relationship with HCAs). The facilitator (N.M.) asked HCAs to share their experience with Healthy Moves during the 4-month intervention period. This study focused specifically on HCAs’ discussion of the program’s effects on HCAs themselves. The focus group sessions were audio-recorded and professionally transcribed.

#### 2.5.3. Analytic Strategies

Conventional content analysis and summative content analysis [23,24] were utilized to analyze the qualitative data from the open-ended survey question and the post-intervention focus group with HCAs, respectively. For the open-ended question (“Could you please tell me what makes you feel (that you are very or somewhat satisfied with Healthy Moves?)”), a research assistant (T.L) read through all the responses, identified key themes, and developed a codebook. The focus group data were independently coded by two research assistants. One research assistant (T.L.) analyzed the transcripts specifically to search for the two themes related to this current study: “HCAs did Healthy Moves with their client” and “Healthy Moves changed HCAs’ health behavior”. T.L. examined the identified quotes further and developed sub-themes. The other research assistant had previously coded all the focus group transcripts for other purposes. When there was disagreement between the two sets of the codes with respect to the two themes for the current study, the principal investigator (N.M.) joined the discussion. Final decisions about the codebooks were made in consensus for both the open-ended survey questions and the post intervention focus groups with HCAs. Using the final codebooks, T.L. coded both the open-ended survey and the focus group data and summarized findings. 

## 3. Results

### 3.1. HCA Participant Baseline Characteristics

As described in Table 1, the 46 HCA participants were typically African American women with a mean age of 49 at baseline. They typically completed high school education or some college, were not married currently, and were living with others. Nearly half of them had multiple chronic conditions, 30% had Medicare, 48% had Medicaid, and 22% had both Medicare and Medicaid. On average they had worked as a HCA for 6.5 years (78 months), and about 22% had other paying jobs.

### 3.2. Did HCAs’ Own Physical Activity Increase after the Intervention?

Table 2 summarizes changes in HCAs’ physical activity levels and related variables between the baseline and post-intervention assessments. Older HCAs aged 50+ reported they spent more time doing the types of physical activity (stretching or strengthening) that they delivered to their clients (*p* = 0.027). However, no such increase was observed among younger HCAs. There was no statistically significant change in time spent on other types of physical activity nor in readiness to exercise regularly and exercise self-efficacy in HCAs.

**Figure 1:**
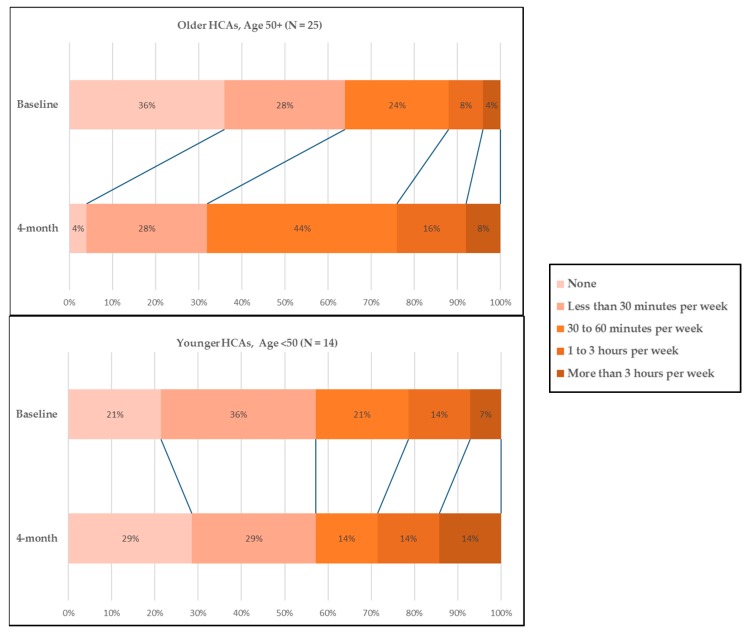
Home care aides’ stretching or strengthening time spent in the last week.

To follow up on the significant finding that older HCAs increased time spent on stretching or strengthening, we graphically depicted changes in time spent on stretching or strengthening exercises between the baseline and Month 4 among older and younger HCAs. Figure 1 clearly indicates that time spent on this type of exercise increased in older HCAs, but not in younger HCAs. The percentage of older HCAs (50+) who spent 30 min or more per week on stretching or strengthening exercise increased from 36% to 68%, while those who did not do such exercise declined from 36% to 4%. Out of 25 HCAs aged 50+, nearly half (*N* = 12) increased time spent on stretching or strengthening exercise. In particular, older HCAs who reported “none” at pre-intervention assessment reported spending up to 30 min (*N* = 1), 30–60 min (*N* = 4), 1–3 h (*N* = 3), and more than 3 h (*N* = 1) weekly on stretching or strengthening exercise.

**Figure 2:**
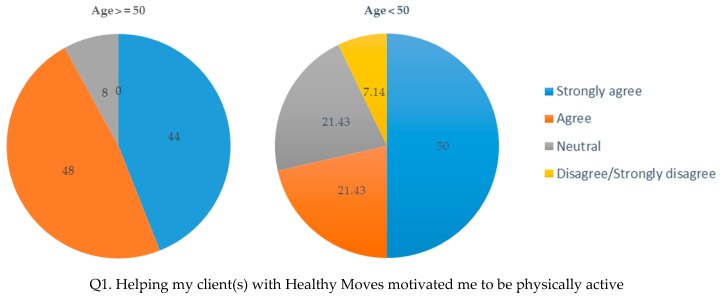

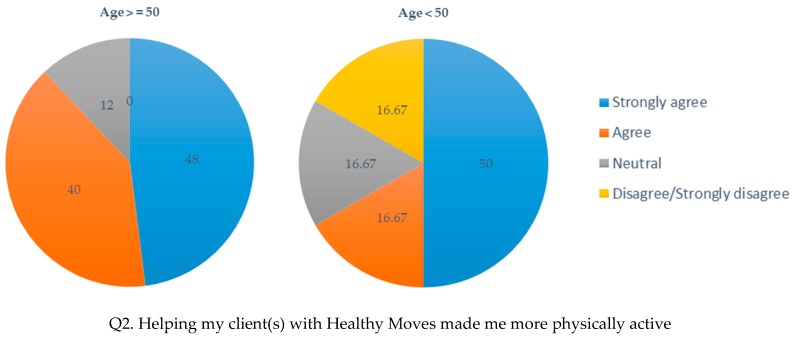
HCAs’ perception of how Healthy Moves impacted their own physical activity (percent).

The post-intervention survey data corroborated the above findings among older HCAs. As shown in Figure 2, older HCAs were more likely than younger HCAs to “strongly agree” or “agree” that helping clients with Healthy Moves motivated HCAs to be physically active (92% vs. 71%) and made them more physically active (88% vs. 67%).

Focus group data revealed that some HCAs did Healthy Moves with their clients. Twelve of the 13 HCAs who mentioned doing Healthy Moves were aged 50 or older (range: 51–73). Qualitative data analysis provided several themes about HCAs’ doing Healthy Moves with their clients. The most frequently mentioned theme was that HCAs felt they benefited from Healthy Moves themselves, as expressed by one HCA:

“…if it helped me it had to have helped her... I did it with my client… We’ll sit there and do them together… And I was like whoa. I can feel that.”

Another frequently mentioned theme was to help clients. For example, one HCA said:

“She (the client) is a diabetic, and she’s on the dialysis machine….. Sometimes she tells me, ‘I’m tired.’ So I say, ‘OK. We gonna get some rest.’ And then she’ll say, ‘we gonna do one more with the feet.’ I say, ‘OK’. So I let her, you know, go at her pace.”

Some HCAs mentioned that they do Healthy Moves with clients to motivate them, as shown in the following quote:

“….when I come in, he (the client) tells me ‘now we don’t need to do it (Healthy Moves)’, I say, ‘yeah, we gonna do it together.’”

The benefit of Healthy Moves for the client sometimes leads HCAs to do Healthy Moves together:

“My client is a 90-year old gentleman and he loves Healthy Moves. He reminds me most of the time. ….In fact, it helps him and it helps me as well because I always do it with him.”

Interestingly, some HCAs were motivated by their clients who were enthusiastic about Healthy Moves.

“….when we did the arm curls….she (the client) got me beat. Because you know after so long, my arms gonna get tired, but she just keep goin’.”

One of the older HCAs reported doing Healthy Moves with her clients via FaceTime using her i-Pad:

“You got time to do Healthy Moves now? I say ‘OK, let me get to the computer. We do it FaceTime.’ She get on her laptop FaceTime, because we both have iPads, so we FaceTime each other.”

### 3.3. How Was Healthy Moves Received by HCAs?

Overall, Healthy Moves was well-received by HCAs. As shown in Table 3, most HCAs “strongly agreed” or “agreed” that the Healthy Moves training increased their ability to motivate clients to be physically active (90%), their ability to help clients with safe exercise (95%), and their ability to communicate with their clients (90%). They felt their knowledge about physical activity increased (95%). HCAs agreed that they have used what they learned from the program for their clients (95%) as well as their family or friends (85%).

Ninety-eight percent of HCAs were satisfied with the program. Five major reasons for their satisfaction emerged: (1) client likes the program, (2) program benefits clients, HCAs, or both, (3) program makes the HCA helpful for their client(s), (4) program helps improve the HCA’s relationship with the client and (5) HCA was happy to see the client move around and do the program the HCA delivered. One of the HCAs responded:

“Every time I ask my aunt if she did her exercise, her face lights up and even when she talks about it to the doctor.”

The most frequently mentioned reason why HCAs were satisfied with conducting Healthy Moves with their clients was that it was beneficial for both the clients and the HCAs themselves. One of the HCAs responded:

“Healthy Moves reminded me that I did not have to leave (the home) to engage in physical activity, that I needed to make time every day for my health. And I appreciate the past 4 months on a personal level.”

The following comments highlight Healthy Moves’ benefits:

“It made my clients more motivated and wanting to do the exercises without any help. It also increased their strength as far as walking and balancing better.”

“…I used to have (a) hard time exercising because of arthritis, but now I'm better and do more.”

The program’s dual benefits for clients and HCAs were expressed by one HCA:

“It is very satisfying because the client (was) trying Healthy Moves and me, too. It gives us both energy to start the day.”

The program also provided the chance for their clients to do something and helped HCAs’ relationship with their clients:

“It helps the client’s health, mood, and allows for a better bond with client...”

Some HCAs commented that Healthy Moves helped their health problems, such as having poor circulation in their arms. Some of the HCAs stated that Healthy Moves inspired them to increase their level of exercise or to walk. One of them said, “I started the Healthy Moves, …if I need to lose weight, I’m gonna lose weight… so I’m walking a lot more than I was. My thing was walking…”

#### Another HCA mentioned:

“(My client) has arthritis really bad. And it’s really helped her. She used to get the shots. But since she’s been, we’ve been doing this; she hasn’t had to have it… And she was glad too. Actually she was the one that signed up for it and told me about it…. So that got me to even, you know, exercise more.”

#### An HCA in her 30’s mentioned that she would like to keep doing Healthy Moves as she gets old.

Finally, one HCA said,

“I give my whole family Healthy Moves, because it helps us and we benefit from it.”

## 4. Discussion

This study demonstrated that building health promotion into the work of HCAs was feasible and effective in promoting physical activity in HCAs, especially among older HCAs. Results from quantitative analysis of pre- and post-intervention HCA surveys indicated that older HCAs spent more time on physical activity (stretching or strengthening) after the intervention. The program was well received by HCAs. The safe, gentle physical activity program delivered by HCAs helped create a positive working environment for most HCAs. Many HCAs perceived the program as fun and beneficial. One of the HCAs stated that her client enjoyed the Healthy Moves program so much that the HCA was motivated to do Healthy Moves with the client. Healthy Moves was instrumental in building HCAs’ relationships with their clients and in motivating HCAs on their jobs. Our mixed methods approach allowed triangulation of results from quantitative survey data and qualitative data (open-ended survey questions and focus groups) and revealed improvements in HCAs’ psychosocial work environment during the pilot intervention.

Our study had limitations. The single group pre-post study design limited our ability to infer causality, and the small sample size did not allow multivariate analysis. Self-reported measures of HCAs’ physical activity are likely to have introduced recall and social desirability biases. Our mixed methods approach partly addressed those limitations. For example, the qualitative data helped us understand the context that produced the quantitative results, indicating how our intervention program led to increased physical activity among older HCAs. Our positive results warrant a larger randomized controlled trial for testing the effectiveness of our program for HCAs as well as for their clients. Future research should include objectively measured physical health as well as measures of mental health among HCAs to investigate the program’s additional effects on psychosocial work environment. 

This current study contributes to the small but growing literature on intervention programs that aim to improve the health and safety of home care workers, incorporating life style factors such as physical activity [3,25]. Our study was innovative because of our focus on a health promotion intervention that helped align interests of employers, HCAs, the labor union, community organizations, and the governmental agency that was the payer of the in-home services for older adults. Our project also sought to enhance HCAs′ capacities to promote health and safety for themselves, their clients and their communities. In fact, this program delivered by HCAs led to improvement in their clients’ function measured subjectively and objectively (i.e., fitness tests), as reported elsewhere [26]. This supplementary finding demonstrates our study’s additional impact for the beneficial role of HCAs.

Our research challenges the current home care work paradigm of a “passive care model” where HCAs carry out care plans to help older adults. Our program aims to develop a new work environment for home care workers, which would facilitate a cultural shift towards a model where HCAs and their clients work together on a safe physical activity program that benefits both HCAs and their clients. We envision a home care model that empowers HCAs (and other direct care workers who work in health care and similar service industries) to contribute to the service recipient’s care team through health promotion, not just housekeeping and personal care services.

## 5. Conclusions

Workplace wellness programs that are popular in large companies and certain sectors are not available for the predominantly female direct care workforce [12]. Building health promotion into the job is a promising concept for occupations involving clients, including other direct service workers such as nursing assistants, community health workers and child care workers. Intervention programs that target direct service workers could target clients as well as workers. Such programs could align interests of employers and workers and have a higher likelihood of employers’ adoption than a program focused solely on workers. Our current study demonstrated the feasibility of incorporating this concept into a health promotion program delivered by HCAs for their clients. Our research also indicated promising results of our program among HCAs who were aged 50 or older. Future research should test our program in larger study samples with rigorous research methods. It will be critical to consider workers’ age, socioeconomic and cultural contexts, and life course to develop the most effective health promotion programs for these direct care workers who have both challenges and great untapped potential.

## Figures and Tables

**Table 1:** Baseline characteristics of home care aides.

Variables	All Home Care Aides	Home Care Aides in Pre and Post	Focus Group Participants
	Percent or Mean (Range) *N* = 46	Percent or Mean (Range) *N* = 41	Percent or Mean (Range) *N* = 31
Age, years ^a^	49 (22–73)	49 (22–73)	50 (30–73)
Female	91	90	90
African American	93	98	97
Education			
Below high school	9	10	10
High school or passing a high school equivalency test	28	27	29
Some college or trade school	54	56	55
College and above	9	7	6
Not married currently	85	85	84
Live alone	20	22	19
Number of chronic conditions	2 (0–6)	2 (0–6)	2 (0–6)
2 or more chronic conditions	48	49	55
Health insurance			
Medicare only	9	7	29
Medicaid only	26	27	42
Medicare & Medicaid	22	22	19
Family HCA ^b^	46	49	55
HCA tenure (months)	78 (1–344)	83 (1–344)	80 (1–300)
Has multiple jobs ^c^	22	23	23

^a^ All: *N* = 44; Focus group: *N* = 30; Interview: *N* = 9; ^b^ Family HCA: A home care aide who is employed by the home care agency to care for a member of his or her family; ^c^ All: *N* = 45; Focus group: *N* = 30; Interview: *N* = 10.

**Table 2:** Home care aides’ physical activity: Before and after 4-month intervention ^a^ (*N* = 41).

Variables	All (*N* = 41)			Age ≥ 50 (*N* = 25) ^b^			Age < 50 (*N* = 14)		
	Pre	Post	*p*	Pre	Post	*p*	Pre	Post	*p*
Physical activity time ^c^									
Stretching/strengthening	2.32	2.73	ns	2.16	2.96	0.027 *	2.57	2.50	ns
Walking	3.41	3.34	ns	3.36	3.36	ns	3.50	3.50	ns
Swimming ^d^	1.18	1.15	ns	1.29	1.08	ns	1	1.29	ns
Bicycling	1.66	1.29	ns	1.68	1.48	ns	1.71	1	ns
Using equipment other than dumbbells ^e^	1.44	1.49	ns	1.30	1.55	ns	1.57	1.36	ns
Other ^f^	1.36	1.42	ns	1.4	1.4	ns	1.33	1.5	ns
Readiness to exercise regularly ^g^	3.87	3.92	ns	4.13	4.22	ns	3.46	3.46	ns
Exercise self-efficacy ^h^									
Sticking to it	3.87	3.87	ns	3.92	4.10	ns	3.81	3.47	ns
Making time for physical activity	3.60	3.54	ns	3.66	3.69	ns	3.48	3.34	ns

* *p* < 0.05, ns = not significant; ^a^ Means were reported for each item. *p* values were produced by Wilcoxon matched-pairs signed rank tests for physical activity time and readiness to exercise regularly. *p* values for exercise self-efficacy were produced by paired *t*-tests; ^b^ Two HCAs did not provide birthdate; ^c^ Total time spent on each type of exercise in the past week. 1 = none, 2 = less than 30 min, 3 = 30–60 min, 4 = 1–3 h, and 5 = more than 3 h; ^d^ All: *N* = 40; Age d ≥ 50: *N* = 24; ^e^ All: *N* = 39; Age ≥ 50: *N* = 23; ^f^ All: *N* = 33; Age ≥ 50: *N* = 20; Age < 50: *N* = 12; ^g^ “Do you exercise regularly?” 1 = No, and I do not intend to in the next 6 months, 2 = No, but I intend to in the next 6 months, 3 = No, but I intend to in the next 30 days, 4 = Yes, I have been for less than 6 months, and 5 = Yes, I have been for more than 6 months; ^h^ Participants rated their confidence in exercise habit items on a 5-point scale (1 = I know I cannot, 3 = maybe I can, 5 = I know I can). An average score was calculated for “Sticking to it” (8 items), and “Making time for physical activity” (4 items).

**Table 3:** Home care aides’ evaluation of Healthy Moves program (*N* = 41).

**Questions**	**Strongly Agree %**	**Agree %**	**Neutral %**	**Disagree/Strongly Disagree %**
The training increased				
my ability to motivate my client to be physically active	56.1	34.1	9.8	0
my ability to help clients with safe exercise	51.2	44.0	2.4	2.4
my ability to communicate with my client(s)	53.7	36.6	7.3	2.4
my knowledge about physical activity	61.0	34.1	4.9	0
I have used what I learned for				
my client(s)	58.5	36.6	4.9	0
my family or friends	46.4	39.0	12.2	2.4
**Questions**	**Very Satisfied**	**Somewhat Satisfied**	**Somewhat Dissatisfied**	**Very Dissatisfied**
Overall, how satisfied are you with Healthy Moves for Aging Well?	82.9	14.7	0	2.4
